# Potential Regulatory Interactions of *Escherichia coli* RraA Protein with DEAD-box Helicases* [Fn FN2]

**DOI:** 10.1074/jbc.M113.502146

**Published:** 2013-09-17

**Authors:** Zbigniew Pietras, Steven W. Hardwick, Szymon Swiezewski, Ben F. Luisi

**Affiliations:** From the ‡ Department of Biochemistry, University of Cambridge, Tennis Court Road, Cambridge CB2 1GA, United Kingdom and; § Institute of Biochemistry and Biophysics, Polish Academy of Sciences, 02-106 Warsaw, Poland

**Keywords:** Analytical Ultracentrifugation, Protein-Protein Interactions, RNA-binding Protein, RNA Helicase, X-ray Crystallography, Regulator of Ribonuclease Activity A, RhlB, RraA, SrmB

## Abstract

Members of the DEAD-box family of RNA helicases contribute to virtually every aspect of RNA metabolism, in organisms from all domains of life. Many of these helicases are constituents of multicomponent assemblies, and their interactions with partner proteins within the complexes underpin their activities and biological function. In *Escherichia coli* the DEAD-box helicase RhlB is a component of the multienzyme RNA degradosome assembly, and its interaction with the core ribonuclease RNase E boosts the ATP-dependent activity of the helicase. Earlier studies have identified the regulator of ribonuclease activity A (RraA) as a potential interaction partner of both RNase E and RhlB. We present structural and biochemical evidence showing how RraA can bind to, and modulate the activity of RhlB and another *E. coli* DEAD-box enzyme, SrmB. Crystallographic structures are presented of RraA in complex with a portion of the natively unstructured C-terminal tail of RhlB at 2.8-Å resolution, and in complex with the C-terminal RecA-like domain of SrmB at 2.9 Å. The models suggest two distinct mechanisms by which RraA might modulate the activity of these and potentially other helicases.

## Teaser

**Background:** DEAD-box helicases in bacteria play a key role in cellular RNA metabolism.

**Results:** The trimeric protein RraA binds to *Escherichia coli* DEAD-box proteins.

**Conclusion:** The mechanism of interaction between RraA and SrmB is shown by x-ray crystallography.

**Significance:** Structural basis of potential regulation of a bacterial DEAD-box helicase.

## Introduction

RraA^3^ (Regulator of ribonuclease activity A) is a ring-shaped homotrimeric protein with the ability *in vitro* to influence the activity of the essential *Escherichia coli* ribonuclease, RNase E (1). As an inhibitor of RNase E, RraA has widespread effects on transcript levels in *E. coli* (2), although its physiological role in ribonuclease regulation is debated. Surprisingly, *in vitro* RraA does not inhibit the catalytic activity of RNase E directly, but appears to act indirectly by occluding RNA binding regions in the C-terminal domain of the ribonuclease (1). These RNA binding domains are adjacent to a site that recruits the DEAD-box helicase RhlB, in a multienzyme assembly known as the RNA degradosome. As a component of the RNA degradosome, RhlB contributes to mRNA decay and RNA processing (3–5). We have previously shown that RraA can directly interact with RhlB, and it is possible that RraA plays two distinct roles in modulating the degradosome, by inhibiting both its ribonuclease and helicase activities (1).

In the Górna *et al.* (1) study, it was shown that RraA is able to interact *in vitro* with two other DEAD-box proteins from *Escherichia coli*, namely RhlE and SrmB. SrmB is one of the five DEAD-box proteins in *E. coli* and is known to contribute to ribosome biogenesis along with CsdA (DeaD), RhlE, and DbpA (6). SrmB targets 23S rRNA *in vivo* and forms a ribonucleoprotein complex with ribosomal proteins L4 and L24 (7). SrmB is believed to act as a chaperone by preventing 23S rRNA structures from misfolding during ribosome assembly, preventing their spurious interaction with 5S rRNA (6, 8). It also helps to prevent interactions between rRNA decay intermediates and nascent 50S ribosome subunits (9).

Although RhlB is the canonical helicase component of the RNA degradosome, other DEAD-box helicases may be recruited into the assembly depending on growth conditions. For instance, under conditions of cold stress, CsdA may be recruited to the degradosome (10), and SrmB becomes associated during stationary growth phase (11). Moreover, the functional interplay between helicases and RNase E may be important *in vivo*, as genetic screens show that mutations of CsdA suppress the phenotype of RNase E defects (12).

Here we further investigate the interaction between RraA and the DEAD-box helicases of *E. coli*. We present biochemical and structural data to characterize the interaction between RraA and SrmB, and provide structural data on the interaction of RhlB with RraA and compare the two complexes. Our data show that RraA can modulate SrmB activity and provide structural insight into the mechanism. Finally, we elaborate on possible functions of the interaction between RraA and DEAD-box helicases.

## EXPERIMENTAL PROCEDURES

### Protein Purification

*E. coli* BL21(DE3) transformed with protein expression vectors were grown at 37 °C in 2x YT medium (Formedium) supplemented with carbenicillin (100 μg/ml) or kanamycin (50 μg/ml). Expression was induced by addition of 1 mm isopropyl β-d-thiogalactopyranoside at an *A*_600 nm_ of 0.5. Cells were harvested after 3 h by 4200 × *g* centrifugation at 4 °C for 30 min and pellets were re-suspended in lysis buffer (50 mm Tris/HCl, pH 8.0, 100 mm NaCl, 50 mm KCl, 5 mm MgCl_2_, Complete EDTA-free Protease Inhibitor Mixture (Roche Applied Science)). In the case of CsdAΔ, the lysis buffer was supplemented with 300 mm NaCl. Cells were lysed with an Emulsiflex-05 cell disruptor (Avestin) and the lysate clarified by centrifugation (30,000 × *g* at 4 °C for 30 min). Molar extinction coefficients (ϵ_280_, cm^−1^ m^−1^) were calculated with ProtParam. Nucleic acid contamination was estimated photometrically. All protein samples used were free of nucleic acid contamination.

#### 

##### RhlE, SrmB, SrmB(1–394), and DbpA

RhlE, SrmB, SrmB(1–394) (13), and DbpA (14) were precipitated from soluble cell lysate with ammonium sulfate (60% saturation for RhlE, SrmB, DbpA; 40% for SrmB(1–394)). Pellets were harvested by centrifugation, resuspended in heparin loading buffer (50 mm Tris/HCl, pH 7.5, 50 mm NaCl), and loaded onto a HiTrap heparin HP column (GE Healthcare). Proteins were eluted with a linear gradient to 100% heparin load buffer supplemented with 2 m NaCl, and the purified proteins were pooled and stored at −80 °C until required.

##### CsdAΔ

Vector pROEX-HT encoding a truncated CsdA (CsdAΔ) with N-terminal His tag was a generous gift from Dr. M. Dreyfus and Dr. T. Bizebard (CNRS, University Paris, Institut de Biologie Physico-chimique, France). Clarified lysate from induced cells was loaded onto a HisTrap FF column (GE Healthcare), washed with buffer containing 50 mm Tris/HCl, pH 7.5, 150 mm NaCl, 50 mm (NH_4_)_2_SO_4_ and a gradient was developed to 100% wash buffer supplemented with 250 mm imidazole. Collected fractions were supplemented with 2 mm DTT and 2 mm EDTA, dialyzed against storage buffer (20 mm Tris/HCl, pH 7.5, 500 mm NaCl, 10 mm DTT) and stored at −80 °C.

##### RhlB(398–421) Peptide

A peptide corresponding to residues 398–421 of RhlB with an additional tyrosine to enable photometric estimation of peptide concentration was synthesized by EZBiolab Inc. (YRLTRPRTGNGPRRTGAPRNRRRSG). The peptide was reconstituted in 20 mm sodium phosphate buffer, pH 7.5.

##### RraA

RraA was expressed and purified as described previously (1).

### Analytical Ultracentrifugation (AUC)

Sedimentation velocity experiments were performed using an XL-I analytical ultracentrifuge (Beckman), An60-Ti (Beckman) rotor, and absorbance optics. Absorbance was monitored at 280 nm. The centrifugation was performed at 45,000 × *g* and 20 °C with scans every 2 min. All proteins used in this set of experiments were exchanged into the following buffer: 150 mm NaCl, 50 mm HEPES, pH 7.5, 10% glycerol (w/v). For experiments involving CsdAΔ the buffer contained 300 mm NaCl and 2 mm tris(2-carboxyethyl)phosphine as reducing agent. The mixtures were at 1:1 ratio of helicase:RraA trimer. The sample volume was 400 μl and the concentration was adjusted so the *A*_280 nm_ was between 0.8 and 1.0, corresponding to final protein concentrations of ∼10 μm. Recorded data were analyzed using SEDFIT software (15). Buffer density, viscosity, and partial specific volumes were calculated using SEDNTERP (16). The profiles and fits are presented in supplemental Figs. S2–S6.

### Small Angle X-ray Scattering (SAXS)

SrmB and its complex with RraA proteins were dialyzed at 4 °C against buffer containing 150 mm NaCl, 20 mm HEPES, pH 7.0, 5% (v/v) glycerol, 2 mm DTT. SAXS data were collected at the Deutsches Elektronen-Synchrotron (DESY, Hamburg, Germany) at beamline X33. Scattering profiles were recorded at 15 °C for various concentrations of protein samples (5 to 55 μm). Data were analyzed using the ATSAS package (17). Solvent scattering measurements were averaged and subtracted from the sample data. The radius of particle gyration (*R_g_*) was estimated from the scattering profile at small angle (|*s*| <1.3/*R_g_*) using the Guinier approximation (18). GNOM software (19) was used to estimate maximum particle diameter (D_max_) and calculate the distance distribution function P(*r*). *Ab initio* modeling was performed using DAMMIF (20), which reconstructs the shape of a particle using dummy beads in a sphere of diameter equal to D_max_. DAMMIF reconstructions were performed using the University of Cambridge CamGrid computing cluster (21). Multiple independent reconstructed shapes were aligned, superimposed, averaged, and filtered using DAMAVER and SUBCOMB (22). The quality of averaged shapes was evaluated by the normal spatial discrepancy value (23), which measures dissimilarity between the individual reconstructed shapes. Normal spatial discrepancy values greater than 0.7 are an indication of an unstable solution (22), and the value for the SrmB-RraA complex was 0.62 suggesting a good degree of similarity between individual models.

### Protein Crystallization

SrmB and RraA were mixed at a 1:3 molar ratio and concentrated to 8.7 mg/ml. Crystals were prepared using the hanging drop method at 16 °C by mixing 1 μl of the sample with 1 μl of mother liquor (100 mm magnesium acetate, 100 mm MOPS, pH 7.2, and 12% (w/v) polyethylene glycol 8000). Crystals appeared after 4 weeks but dissolved after a further week. Soon after the crystals appeared they were transferred briefly to reservoir solution supplemented with 25% (v/v) glycerol and flash frozen in liquid nitrogen.

For co-crystallization of RraA with RhlB(398–421) peptide, components were mixed at 1:20 concentration ratio of RraA:RhlB(398–421). The mixture precipitated but could be solubilized at higher ionic strength, resulting in final concentrations of 74.8 μm RraA, 1.48 mm RhlB(398–421), 423 mm NaCl, 99 mm (NH_4_)_2_SO_4_. Co-crystals were obtained using the hanging drop method, by mixing 1 μl of protein sample with 1 μl of mother liquor (100 mm sodium citrate, pH 5.4, 32% methylpentanediol, and 200 mm ammonium acetate) at 16 °C. Crystals were directly flash frozen in liquid nitrogen.

X-ray diffraction data were collected at the Diamond Light Source synchrotron radiation facility (Harwell, UK) at 100 K. The SrmB/RraA crystal diffracted to 2.9 Å, and the RraA/RhlB peptide crystal diffracted to 2.8 Å. The CCP4 suite (24) was used for data processing, molecular replacement, model building, refinement, and other calculations. Data were processed using Mosflm (25) and Pointless and Scala (26). Molecular replacements were performed using Phaser (27) with search models: RraA (PDB code 1Q5X) and Vasa (both domains were used separately with all residues mutated to alanine, PDB code 2DB3). Manual model building was performed using Coot (28). Automated model building and sequence docking was performed using ARP/wARP (29) and Buccaneer (30). Automated refinement was performed using Refmac5 (31). The structures were analyzed by Areaimol, PISA (32), and Superpose (33). The crystallographic and refinement summary are presented in Table 1.

### ATP Turnover Assays

ATPase activity of DEAD-box proteins was monitored using the EnzCheck Phosphate Assay Kit (Invitrogen) following the manufacturer's instructions. All proteins were exchanged into the following assay buffer: 100 mm NaCl, 50 mm Tris/HCl, pH 7.5, with the exception of CsdAΔ, which requires high salt for stability and was exchanged into the assay buffer supplemented with 500 mm NaCl. The final concentrations of DEAD-box helicases were 1 μm except for DbpA, which was used at a final concentration of 2 μm. RraA was added in 3- or 6-fold molar excess over helicase.

Two types of RNA substrates were used in these reactions. The first was a mixture of 16S and 23S rRNA from *E. coli* (Roche Applied Science), for which 2 μg was added to each reaction. The second RNA was a self-complementary 24-mer (5′-GAAUGUACAUCAGAGUGCGCACUC-3′), which anneals to a 12-bp region and has a 12-bp 5′ ssRNA overhang (5) and was used at a final concentration of 1 μm. Reactions were started with the addition of a mixture of ATP and MgCl_2_ (1:1, mol:mol) to a final concentration of 2 mm.

The final reaction volume was 200 μl containing 5 mm DTT and the salt concentration was kept constant at 50 mm NaCl. Absorption at 360 nm was measured in MicroWell 96-well plates (VWR) in a plate reader (Molecular Devices Spectramax Plus) for 900 s and the temperature kept constant at 30 °C. All reactions were performed in triplicate or greater. Absorbance values were converted to the corresponding phosphate concentration using a standard curve. The activity was expressed as moles of inorganic phosphate released per min/mol of protein. The reactions of RhlE and CsdAΔ with 16S and 23S rRNA were characterized by a rapid release of phosphate followed by a plateau phase, therefore a time point of 150 s instead of 900 s was used. Contaminating inorganic phosphate present in the ATP and MgCl_2_ mixture was quantified and subtracted from the results.

### Electrophoretic Mobility Shift Assays (EMSA)

Reactions were in a final volume of 20 μl and included 4 μl of loading buffer: 50% (v/v) glycerol, 0.05% (w/v) bromophenol blue, 50 mm Tris, 384 mm glycine, pH 8.3. Reactions were separated on 5% native polyacrylamide gels (acrylamide:bisacrylamide 37.5:1, 200 mm Tris/HCl, pH 8.5, 10% (v/v) glycerol) in 1× Tris glycine running buffer containing 1 mm DTT at 120 V for 160–180 min at 4 °C. Proteins were exchanged into the following buffers using Micro Bio-Spin 6 columns (Bio-Rad): RraA and CsdAΔ (50 mm HEPES, pH 7.5, 300 mm NaCl, 10% glycerol (w/v), 2 mm tris(2-carboxyethyl)phosphine), and DbpA, RhlE, and SrmB (50 mm HEPES, pH 7.5, 10% glycerol (w/v), 150 mm NaCl). Final helicase and RraA concentrations were 10 and 30 μm, respectively, unless indicated otherwise. For further analysis, selected bands were extracted, incubated with 4× NuPAGE LDS Sample Buffer (Invitrogen) supplemented with 5% (v/v) β-mercaptoethanol for 30 min at 95 °C, then buffer with eluted protein was loaded onto 10% BisTris NuPAGE SDS-PAGE gel (Invitrogen). Subsequent electrophoresis was performed at 180 V for 90–120 min using SDS-MOPS buffer.

### Site-directed Mutagenesis

Site-directed mutagenesis was performed using the Phusion Site-directed Mutagenesis Kit according to manufacturer's instructions (Thermo Scientific).

## RESULTS

### RraA Interaction with SrmB and RhlB

**FIGURE 1.**
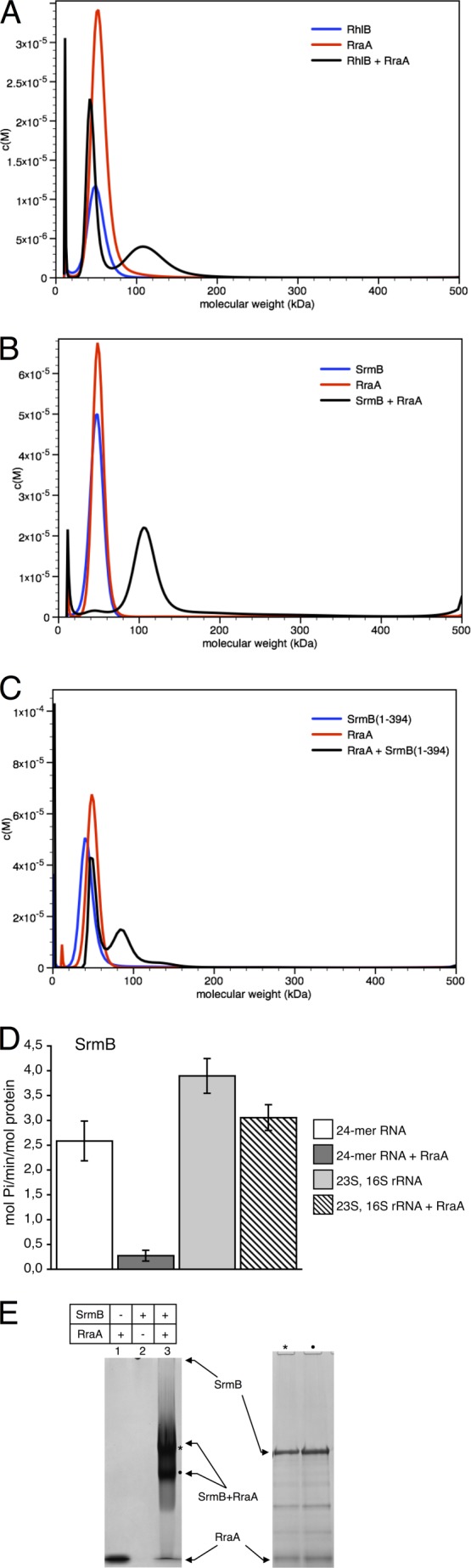
**Stoichiometric and functional analysis of RraA complex with DEAD-box helicases SrmB and RhlB.** The mass distribution for helicases, RraA, and the mixtures are shown in *blue*, *red*, and *black*, respectively. *A–C,* analytical ultracentrifugation of RhlB, SrmB, and SrmB(1–394) in the presence and absence of RraA. *D,* effects of RraA on the ATPase activity of SrmB. *White* and *dark gray bars* represent reactions with 24-mer RNA substrate in the absence and presence of RraA, respectively. Reactions with a mixture of 23S and 16S rRNA in the absence and presence of RraA are represented by *light gray bars* and *bars filled with diagonal stripes*. When present, RraA was added in 3-fold molar excess to helicase. Activity is expressed as mol of P_i_/min/mol of protein. Each *bar* represents averaged values from at least three independent experiments, and *error bars* represent 2 S.D. *E,* interactions of SrmB and RraA evaluated by native polyacrylamide gel elecrophoresis. Free RraA migrates with the buffer front, but due to its positive charge, free SrmB does not enter the gel. The band corresponding to a complex of the two proteins (marked with *asterisk*) was extracted and analyzed by SDS-PAGE, confirming the presence of both RraA and SrmB.

A direct protein-protein interaction has previously been identified between RraA and RhlB. The interaction strength was estimated by surface plasmon resonance to have a dissociation constant of 80 μm, however, EMSA estimate the dissociation constant to be 10 μm (1). To investigate this interaction further we analyzed the RraA-RhlB protein complex using AUC. RhlB and RraA were found to sediment as single species in a molecular mass range close to 50 kDa, corresponding to the expected mass of trimeric RraA (52 kDa) and the monomeric RhlB (47 kDa). When RraA and RhlB were mixed at a 3:1 molar ratio (of protomers), at 10 μm total protein concentration, a faster sedimenting species with apparent mass near 100 kDa was observed (Fig. 1*A*). Similarly, when a mixture of RraA and SrmB was analyzed by AUC at a similar concentration, again a species corresponding to a complex of ∼100 kDa was observed (the SrmB monomer has a molecular mass of 50 kDa). These data suggest RraA is capable of forming a direct interaction with either RhlB or SrmB *in vitro*, and the observed 100-kDa mass for both complexes corresponds to an assembly of one RraA trimer and one helicase monomer.

The interaction of RraA with SrmB was further corroborated by EMSA using non-denaturing polyacrylamide gels (Fig. 1*E*). Bands corresponding to an RraA-helicase complex were excised from the gel, and subsequent analysis by SDS-PAGE confirmed the presence of both helicase and RraA proteins in the single gel band.

When simply comparing the AUC profiles of the complexes of RraA with SrmB or RhlB the data suggests that the affinity of RraA for SrmB is greater than for RhlB. The majority of SrmB and RraA co-sediment in the 100-kDa peak, with little free RraA or SrmB, suggesting the dissociation constant for this interaction is well below 10 μm. However, the RraA/RhlB mixture shows a significant amount of protein remains in the 50-kDa peak, suggesting a weaker interaction (compare Fig. 1, *A* and *B*).

Our previous investigations of the complex between RhlB and RraA showed that this interaction is mediated almost exclusively through the RhlB C-terminal extension (CTE) (1). In the present study we have confirmed by isothermal titration calorimetry a direct interaction between RraA and RhlB CTE (data not shown). Given that the AUC data indicates SrmB forms a similar complex with RraA as RhlB we performed AUC with a truncated SrmB lacking the C-terminal extension (SrmB(1–394)) to establish if this interaction is also mediated through the CTE of the helicase. Surprisingly deletion of the CTE of SrmB does not abolish the interaction with RraA, in contrast to the results with RhlB, indicating that the SrmB core is also involved in this interaction (Fig. 1*C*). Formation of a complex with RraA is somewhat less efficient with the truncated SrmB protein (compare Fig. 1, *B* and *C*), suggesting that the CTE contributes to this interaction, but is not essential for complex formation.

### RraA Modulates RNA-dependent ATPase Activity of SrmB

We have previously shown that the interaction between RraA and RhlB reduces the ATPase activity of the helicase (1). The capacity of RraA to bind to SrmB raises the question of whether this interaction has a similar inhibitory effect. To address this question, we compared rates of ATP turnover by SrmB in the presence and absence of RraA. The proteins were mixed at a 1:1 ratio (helicase monomer:RraA trimer) in line with the stoichiometry predicted from our AUC experiments, and in the presence of an RNA substrate. 23S rRNA has been shown to be a substrate for SrmB (6, 8), and using a mixture of *E. coli* 23S and 16S rRNA, mild inhibition of ATPase activity was observed in the presence of RraA. The effect was much more pronounced with a corresponding concentration (in nucleotides) of a self-complementary 24-mer RNA, which forms a short (12 bp) RNA duplex with 12-bp 5′ overhangs (Fig. 1*D*). The RNA 24-mer is an artificial construct and is likely to be a nonspecific substrate for *E. coli* DEAD-box helicases (34). The results indicate that the formation of a complex with RraA can modulate the ATPase activity of SrmB and that the magnitude of the effect depends on the nature of RNA substrate. A stronger inhibitory effect of RraA in the presence of nonspecific substrates might help to boost the apparent fidelity of the helicase.

### Structural Basis of RraA Interactions with DEAD-box Proteins

#### 

##### Complex between RraA and SrmB

**FIGURE 2.**
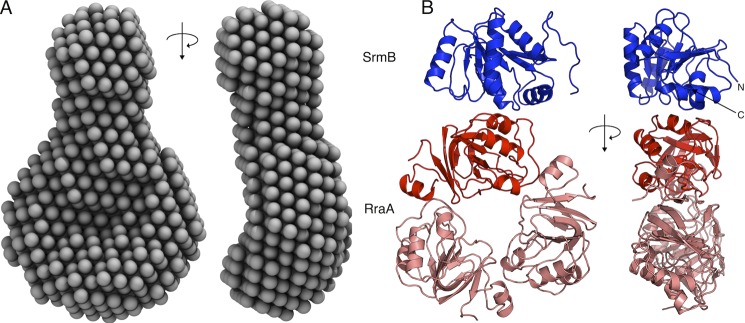
**The solution and crystal structures of the SrmB-RraA complex.** *A,* an averaged *ab initio* SAXS model of SrmB-RraA complex, shown from two viewpoints related by a 90° rotation. *B,* the content of the crystal asymmetric unit (two viewpoints related by a 90° rotation, as in *A*). The RraA trimer is colored *salmon* with the protomer contacting SrmB shown in *red*, SrmB CTD is shown in *blue*. The N and C termini of SrmB are marked by *N* and *C,* respectively.

To obtain insights into how the interaction of RraA with SrmB might influence the activity of the helicase, we obtained solution and crystal structures of this complex. We first performed SAXS to obtain the low-resolution envelope for the complex formed by mixing SrmB and RraA at a 1:3 molar ratio. This envelope can accommodate a single RraA trimer with a single SrmB helicase bound to the outer ring of RraA, consistent with the 1:3 molar ratio indicated by analytical ultracentrifugation analyses (Fig. 2*A*).

We co-crystallized the two proteins and solved the crystal structure at 2.9-Å resolution by molecular replacement using the structures of RraA (35) (PDB code 1Q5X) and a polyalanine model prepared from a single RecA domain of the *Drosophila* Vasa helicase (36) (PDB code 2DB3). The crystallographic asymmetric unit contains a single torus-shaped trimer of RraA bound to one SrmB RecA-like α/β sandwich domain (Fig. 2*B*). For each of the three RraA protomers, residues 2–159 could be modeled, and the entire trimer superimposes with a root mean square deviation of 0.41 Å with the original structure of this protein (35). The crystallographic data and refinement statistics are summarized in Table 1.

**TABLE 1:** **Crystallographic data and refinement summary** Values for highest resolution shell are shown in parentheses. Five percent of the reflections were set aside for calculation of the *R*_free_. Crystallographic and refinement statistics were calculated by Scala (26) and Refmac5 (31), respectively.

	RraA + SrmB	RraA + RhlB(398–421)
Crystallization condition	100 mm MOPS pH 7.2	100 mm Sodium citrate pH 5.4
	12% PEG 8000	MPD 32% (v/v)
	100 mm Magnesium acetate	200 mm Ammonium acetate
Space group	P3_2_21	C2
Cryo-protectant	25% glycerol (v/v)	
Wavelength (Å)	0.9763	0.9795
Resolution (Å)	63.56–2.90 (3.06–2.90)	63.53–2.80 (2.95–2.80)
Unit cell dimensions		
*a*, *b*, *c* (Å)	73.39, 73.39, 222.89	259.63, 69.07, 123.30
α, β, γ (°)	90.00, 90.00, 120.00	90.00, 109.17, 90.00
Completeness (%)	99.6 (99.5)	98.0 (99.3)
Multiplicity	4.0 (4.1)	2.9 (2.9)
*I*/σ	10.2 (2.8)	15.5 (3.7)
*R*_merge_ (%)	12.3 (50.8)	4.9 (25.9)

**Refinement**		
Reflections	15,208	47,500
*R* factor	0.2012	0.2303
*R*_free_	0.2509	0.2900
Bond lengths root mean square deviations (Å)	0.0228	0.0168
*B* factor protein (Å^2^)	42	46
Atoms	4,993	14,445
Residues	673	1970

Following close inspection of the electron density maps it was clear that the RecA-like domain resolved in the crystal structure corresponds to the CTD of SrmB (residues 219–388). The CTD has a typical DEAD-box protein-fold and engages the outside rim of the RraA trimer, such that only one RraA protomer is engaged with the SrmB CTD. There are no significant structural changes in the RraA protomer that contacts the SrmB CTD compared with the two protomers not bound to SrmB. The presence of only the C-terminal RecA-like domain of SrmB in the crystal structure was unexpected. Insufficient protein crystals were obtained to evaluate their protein content by SDS-PAGE, but gel analysis of the entire crystallization droplet suggested that SrmB was intact in solution. However, it is possible that the CTD of SrmB may have been liberated by proteolysis during crystal genesis. The last 56 residues of SrmB corresponding to the CTE, which are predicted to be natively unstructured, were also not resolved in the final model, and this may indicate that this region is indeed disordered. Other DEAD-box proteins have flexible N- and C-terminal extensions that are often not modeled in their crystal structures, with few exceptions (37).

Inspection of the interface between SrmB and RraA reveals electrostatic complementarity formed by a positively charged patch on SrmB and a negatively charged cluster on RraA. The interface involves mainly salt bridging interactions of side chains and hydrogen bonds between main chain atoms. Most of the RraA residues involved in the interaction with SrmB are conserved (supplemental Fig. S1) and two of these residues (Asp-50 and Glu-53) were predicted earlier to be involved in RraA interactions with other proteins (38). In contrast the SrmB residues involved in the interaction lie outside of regions of high sequence conservation in DEAD-box proteins.

**FIGURE 3.**
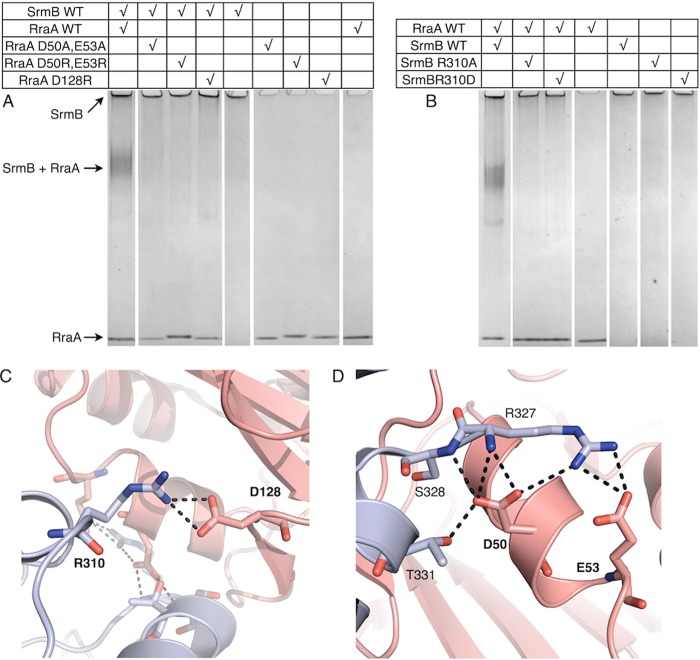
**Analysis of molecular interactions between RraA and SrmB.** *A* and *B,* interactions of WT and mutated proteins evaluated by electrophoretic mobility shift assays. Final SrmB and RraA concentrations were 2.5 and 7.5 μm, respectively. *Panels C* and *D* highlight residues mutated for these analyses. SrmB is *light blue* and RraA is colored *salmon*. For clarity, side chains of residues that do not interact directly with mutated residues are not shown.

The role of the identified interface in stabilizing the complex was tested by site-directed mutagenesis (Fig. 3). Recombinant RraA proteins were generated with a single amino acid mutation at residue Asp-128, and a double mutation of Asp-50 and Glu-53. Similarly, SrmB was expressed and purified with a single mutation at residue Arg-310. The chosen amino acids were substituted for both oppositely charged residues, and alanine. In all cases the substitutions inhibited complex formation as assessed by electrophoretic mobility shift assay.

##### Complex between RraA and RhlB(398–421)

Unlike the interaction between RraA and SrmB, the complex formed between RraA and RhlB appears to be mediated entirely by the CTE of RhlB. Despite extensive efforts we were unable to co-crystallize RraA with full-length RhlB, however, it was possible to obtain co-crystals of RraA with a peptide corresponding to the RhlB CTE (RhlB(398–421)). The structure of RhlB(398–421)/RraA was solved at 2.8-Å resolution, and reveals 12 protomers of RraA in the asymmetric unit of the crystal, organized as stacked trimers that generate two hexamers. The hexameric form of RraA may be a favored oligomerization state at higher protein concentrations, as hexameric assemblies also occur in the crystal structure of isolated RraA (35).

**FIGURE 4.**
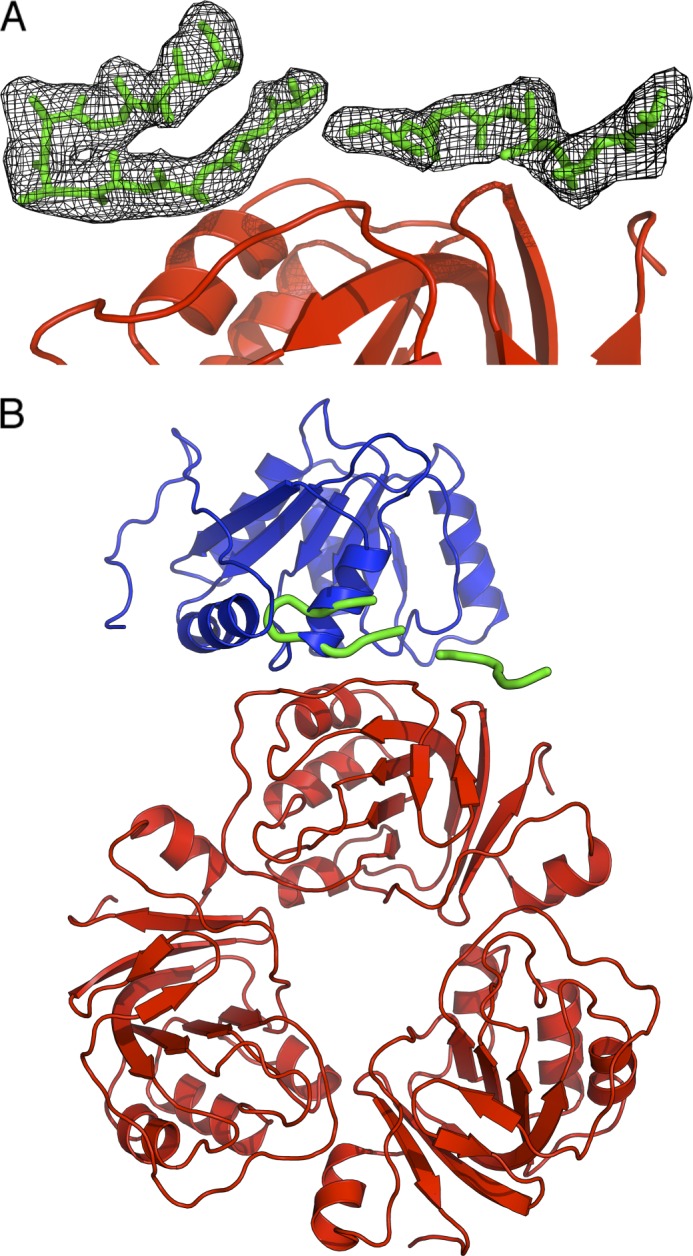
**Crystal structure of RraA in complex with the C-terminal extension of RhlB (RhlB(398–421)).** *A,* the RhlB fragment is shown in sticks representation (*green*). The black mesh represents a 2*F_o_* − *F_c_* electron density map. RraA is colored *red. B,* superposition of SrmB CTD (*blue*) bound to RraA on the RhlB fragment. The RraA trimer is shown in *red*, and RhlB(398–421) is shown in *green*.

The electron density maps were clear and well resolved for all the RraA protomers. Poorer quality electron density was identified on the surface of a single RraA protomer that is able to accommodate a single RhlB(398–421) peptide (Fig. 4*A*). The identified density is likely to originate from RhlB(398–421) as there are no elongated molecules (*e.g.* polyethylene glycol) in the crystallization condition that may account for the continuous density. Due to the poor quality of the density it was not possible to model amino acid side chains for the peptide, but 15 of 23 main chain residues could be traced with confidence (two chains of 6 and 9 residues, Fig. 4*A*). The two segments of the traced main chain likely belong to one molecule; however, the discontinuity in the electron density did not allow the fragments to be joined with confidence. The density is located close to electronegative patches on the RraA surface, which is compatible with the complementary electropositive nature of RhlB(398–421) (predicted pI 12.5). As the peptide side chains could not be modeled unequivocally, it is not possible to derive detailed information about the RhlB(398–421)/RraA interface. Nonetheless, the structure indicates an approximate site of RhlB fragment binding on the surface of RraA. Surprisingly, the contact site overlaps with the interface identified for the SrmB/RraA assembly (Fig. 4*B*) and the modeled RhlB residues lay in proximity of RraA residues Asp-50 and Asp-128. This suggests that RraA harbors one surface region that is the binding site for both RhlB and SrmB.

### Interactions of RraA with Other DEAD-box Enzymes

**FIGURE 5.**
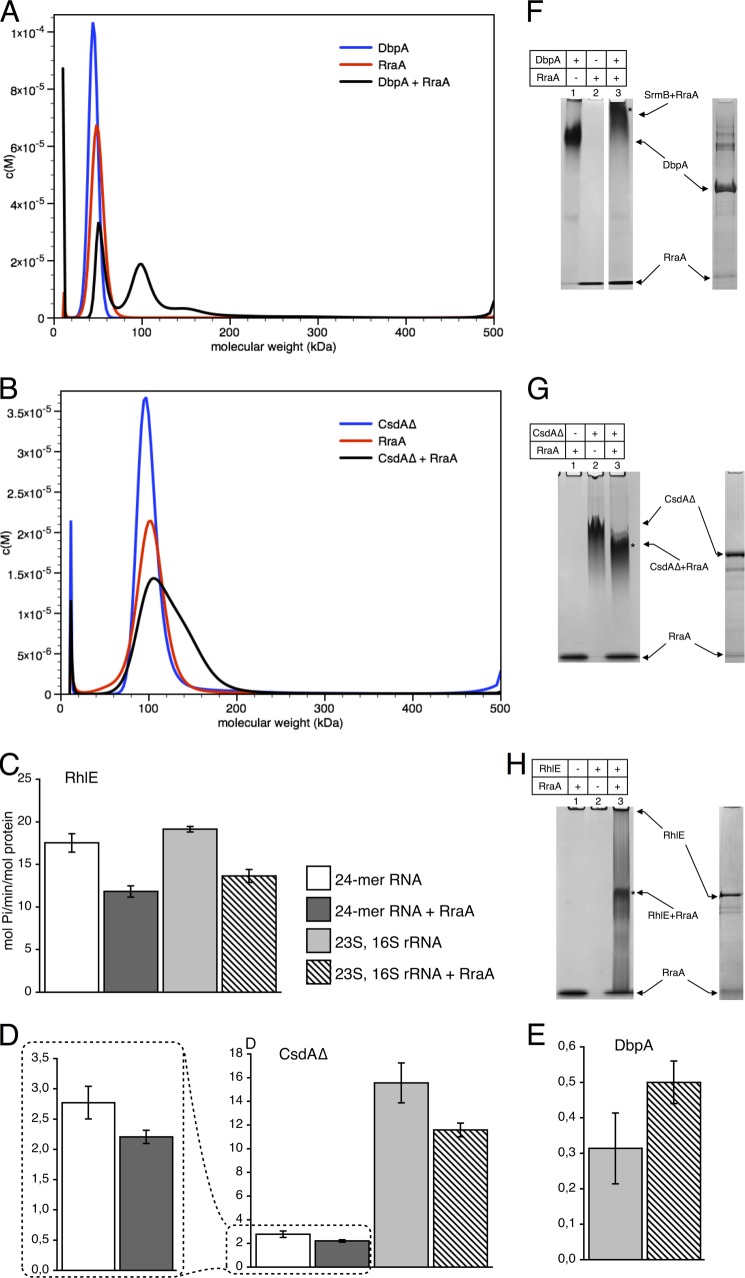
**RraA and its interactions with DbpA, CsdA, and RhlE helicases.** *Panels A* and *B* show AUC analysis. The mass distribution for helicases, RraA, and the mixtures are shown in *blue*, *red*, and *black*, respectively. *A,* DbpA and RraA. *B,* AUC analysis of CsdAΔ, RraA, and their complex (in 300 mm NaCl). Both proteins form particles of ∼100 kDa, which corresponds to a CsdAΔ dimer and RraA hexamer. When both proteins are present a predominant peak is recorded with a shoulder of ∼150 kDa, which likely correspond to an assembly between the CsdAΔ with RraA. *Panels C–E,* effects of RraA on ATPase activity of the helicases. Each *bar* represents averaged values from at least three independent experiments, and *error bars* represent 2 S.D. *White* and *dark gray bars* represent reactions with 24-mer RNA substrate. Reactions with 23S and 16S rRNA are represented by *light gray bars* and bars *filled with diagonal stripes*. Bars that are *dark gray* or *filled with diagonal stripes* represent reactions with RraA. RraA was added in 3-fold molar excess (one RraA trimer per helicase molecule). In the case of CsdAΔ with 23S, 16S rRNA, and DbpA twice the amount of RraA was used (two RraA trimers per helicase molecule). Activity is expressed as mol of P_i_/min/mol of protein, and the scale differs for individual helicases. *Panels F–H,* interactions of RraA, with the DEAD-box helicases analyzed by native PAGE. Free RhlE does not enter the gel. Bands marked with an *asterisk* were extracted and analyzed by SDS-PAGE to confirm the presence of both RraA and DEAD-box proteins.

The findings presented here indicate that RhlB and SrmB use different recognition modes to interact with RraA. We investigated whether RraA might interact with the remaining three DEAD-box helicases encoded by the *E. coli* genome, namely DbpA, CsdA, and RhlE. Notably, AUC and EMSA analysis revealed that all of the DEAD-box proteins studied bind to RraA *in vitro*, albeit to various extents (Fig. 5, *A*, *B*, and *F–H*). In the case of DbpA, AUC revealed a single species in a molecular mass range close to 50 kDa, corresponding to the expected mass of monomeric DbpA (49 kDa). As we have seen for RhlB and SrmB, when DbpA was mixed with RraA a new species was observed with a mass of 100 kDa, which suggests a similar stoichiometry to the RraA complex with SrmB and RhlB (Fig. 5*A*). Interaction of DbpA with RraA was confirmed using EMSA (Fig. 5*F*).

Earlier studies have reported that full-length CsdA has a tendency to aggregate *in vitro*, but a truncated version consisting of residues 1 to 443 (CsdAΔ) was found to have greater solubility, while retaining ATPase activity (39). For the purpose of completeness we included CsdAΔ in our AUC analyses, but appreciate that the removal of the CTE of CsdA may have a detrimental effect on the formation of a complex with RraA. For the AUC analyses of CsdAΔ undertaken here, we found that it was necessary to elevate the NaCl concentration in the buffer to 300 mm to prevent protein precipitation. Under these conditions the AUC profile suggests that CsdAΔ forms a dimer, with a single species observed with an estimated mass of 100 kDa. The same high salt conditions also increased the apparent molecular mass of RraA to ∼100 kDa, which may correspond to hexameric RraA (Fig. 5*B*). The sample with both RraA and CsdAΔ showed a major peak of 100 kDa accompanied by a shoulder of greater mass (∼150 kDa), which may correspond to a complex of both proteins (Fig. 5*B*). However, with these analyses we cannot confidently determine the stoichiometry of the RraA-CsdAΔ complex. Interaction of CsdAΔ with RraA was confirmed using EMSA (Fig. 5*G*).

The final *E. coli* DEAD-box helicase we investigated was RhlE. However, when RhlE was mixed with RraA the solution rapidly precipitated, and consequently it was not possible to confirm the formation of an RhlE-RraA complex by AUC. We were, however, able to confirm the interaction of RhlE with RraA using EMSA (Fig. 5*H*).

Next we investigated if the identified interactions with RraA can also influence the activities of DbpA, RhlE, and CsdAΔ using 23S rRNA or a synthetic 24-mer RNA substrate. The results revealed moderate effects of RraA on ATPase activities compared with SrmB (Fig. 5, *C–E*). We also did not observe a clear dependence on RNA substrate, in contrast to our observations with SrmB.

It is interesting to note that the ATPase activity of DbpA, CsdAΔ, and RhlE were all enhanced in the presence of *E. coli* 23S and 16S rRNA, and all but DbpA were activated by the 24-mer RNA substrate. The results are consistent with earlier findings that DbpA specifically requires 23S rRNA to be active (40). The results also agree with reports that RhlE exhibits the greatest ATPase activity of the *E. coli* DEAD-box helicases (Fig. 5*C*) (39).

## DISCUSSION

Here we have provided structural details of the interaction between RraA and the DEAD-box helicases RhlB and SrmB. The SrmB/RraA crystal structure is in good correspondence with the shape reconstructed from SAXS data and with the stoichiometry suggested by AUC. SrmB interacts on the outside rim of the RraA trimer torus and not with the negatively charged grooves present at the protomer interfaces as previously suggested (1). Our structural data suggest that a common surface on RraA is used for the interactions with RhlB and SrmB. The biological role of this interaction, and additionally the interactions we have observed with other *E. coli* DEAD-box proteins *in vitro* will require further *in vivo* investigations. Our enzymatic assays suggest that RraA is capable of modulating the ATPase activity of these conserved enzymes, and this is likely to have functional consequences if the interactions also occur *in vivo*.

Our *in vitro* enzymatic assays were performed in the micromolar concentration range, and the dissociation constants for the helicase/RraA interactions are likely to be low micromolar. It is estimated that there are roughly 1350 molecules of RhlB, 1000 of DbpA, and 500 to 1000 of RraA in an *E. coli* cell (41–43),^4^ and these values would correspond to micromolar concentrations. Accordingly, binding of RraA and the helicases is expected to occur *in vivo* if the molecules have mutual access.

**FIGURE 6.**
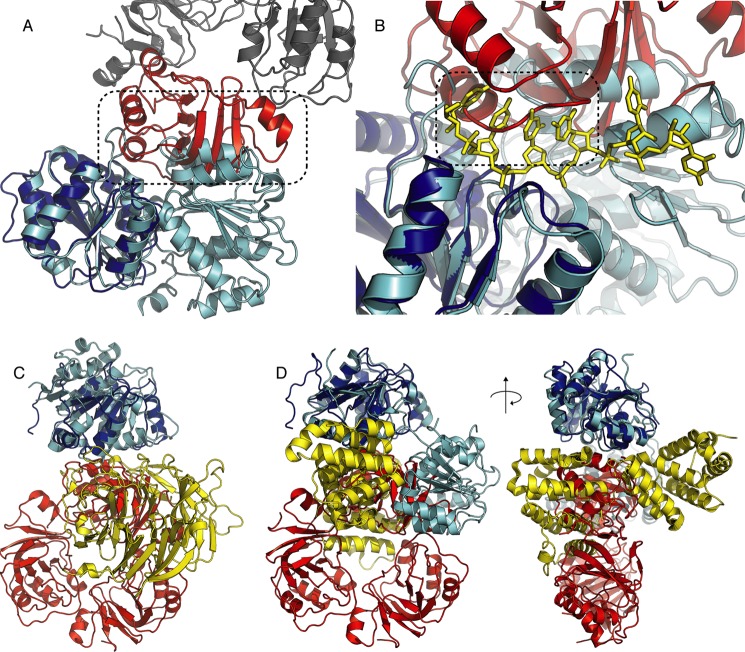
**Comparison of the RraA/SrmB structure and other DEAD-box protein complexes.** *A, B* RraA bound to SrmB would prevent RecA-like domain closure due to steric clash (highlighted by *dashed frame*). SrmB is colored *blue*, the RraA protomer interacting with SrmB is shown in *red* and the other RraA protomers are shown in *gray*. The Dbp5 structure in closed conformation (PDB code 3FHT), superimposed onto SrmB CTD is shown in *cyan* with RNA colored *yellow* and shown in stick representation. The root mean square deviation for the fit is 1.34 Å. *B,* RraA binding may impede RNA binding by SrmB. The RraA loop consisting of residues 53–59 would occlude the predicted RNA binding surface of SrmB (highlighted by *dashed frame*). *C,* comparison of the RraA-SrmB and Dbp5-NUP214 complexes. The reference frame for the overlay is the SrmB CTD (*blue*) and the Dbp5 NTD (*cyan*). RraA is *red* and NUP214 is *yellow* (PDB code 3FHC). *D,* comparison of the RraA-SrmB and eIF4A-PDCD4 complexes. Superposition of SrmB CTD (*blue*) on eIF4A NTD (*cyan*). PDCD4 is shown in *yellow* (PDB code 2ZU6). The overlays are shown from two viewpoints related by a 90° rotation.

Based on our structural data, we propose a mechanism to explain how RraA impedes SrmB activity. Superposition of the SrmB-RraA complex structure onto the crystal structure of the human DEAD-box helicase Dbp5 in complex with RNA and an ATP analog (PDB code 3FHT) reveals that bound RraA would prevent SrmB from adopting the closed conformation seen for the Dbp5 structure (Fig. 6*A*). It is well known that a closed helicase conformation is required for ATP turnover and RNA unwinding (44). Closer inspection of the superimposed helicase structures reveals that bound RraA partially occludes the RNA binding surface of SrmB. If RNA were to be bound to SrmB in a similar manner to the interaction seen for Dbp5, it would clash sterically with a loop on the surface of RraA consisting of residues 53–59 (Fig. 6*B*). Based on these structural overlays, we propose that RraA can influence SrmB activity by sterically inhibiting substrate binding and preventing the enzyme from adopting the closed conformation required for ATP hydrolysis.

Similar mechanisms of inhibiting DEAD-box proteins have been described previously. For instance, in the crystal structure of the eukaryotic DEAD-box helicase Dbp5 in complex with NUP214 nucleoporin (PDB code 3FHC) the N-terminal RecA-like domain of Dbp5 contacts the N-terminal region of NUP214, and this interaction prevents the helicase from adopting a closed conformation and restricts RNA binding (45). It was possible to superpose the CTD of SrmB in complex with RraA onto the NTD of Dbp5 bound to NUP214. This overlay reveals that both protein partners (RraA and NUP214) are bound on equivalent surfaces of the helicase RecA-like domains (Fig. 6*C*). Furthermore, the interaction between Dbp5 and NUP214 is also primarily mediated by surface charge complementarity. However, unlike RraA, NUP214 binds to conserved sequence motifs of the DEAD-box enzyme (45, 46). Another inhibitory partner of DEAD-box proteins is the tumor suppressor programmed cell death protein 4 (PDCD4), which forms a complex with eIF4A helicase (47). However, the mode of binding in this complex is slightly different as PDCD4 engages both domains of eIF4A simultaneously (Fig. 6*D*).

Interestingly the mechanism observed for SrmB seems not to be applicable in the case of RhlB, as this DEAD-box enzyme contacts RraA through an unstructured CTE. As the RhlB C-terminal extension plays a role in nonspecific binding of RNA substrates (5), by binding to the CTE, RraA would compete with nonspecific RNA binding. It was experimentally demonstrated for RhlB that the positively charged CTE is required for ATP turnover in the presence of RNA (13, 48). The importance of the C-terminal extensions of DEAD-box proteins in binding to RNA substrates has recently been highlighted in both Mss116p of *Saccharomyces cerevisiae* and CYT-19 of *Neurospora crassa*, where the CTEs have been shown by SAXS to bind nonspecifically to large RNA substrates (49). In the proposed model for inhibition of RhlB, the RraA would not contact the RNA binding sites located in the helicase core as it does in the case of SrmB, but instead sequester the CTE.

Recently, SrmB was shown to specifically target 23S rRNA, and the helicase has been proposed to prevent formation of misfolded RNA structures during ribosome assembly (6–8). We have seen a much stronger inhibitory effect of RraA in the presence of a nonspecific 24-mer RNA, rather than a specific RNA substrate, *i.e.* 23S rRNA. Thus we hypothesize that a possible function of the RraA interaction with SrmB is to impede the action of helicase on nonspecific substrates. Unsupervised enzyme action could lead to futile expenditure of ATP, misfolded RNA species, and consequently inefficient or erroneous ribosome assembly.

The interaction between RraA and SrmB is a potential link between regulation of RNA levels in the *E. coli* cell and ribosome biogenesis. The RraA protein concentration varies during the *E. coli* growth cycle, increasing more than 5-fold during late logarithmic growth phase (43), and this variation may have impact on helicase activity. Logarithmic phase is characterized by intensive ribosome biogenesis, whereas in stationary phase *de novo* ribosome synthesis is minimal (50, 51). One role of RraA could therefore be to inhibit SrmB activity during transition from logarithmic to stationary growth phase and to exercise control over its specificity.

## Footnotes

4 G. Georgiou, personal communication.

## Footnotes

3 The abbreviations used are:

RraA: regulator of ribonuclease activity A
SAXS: small angle x-ray scattering
BisTris: 2-[bis(2-hydroxyethyl)amino]-2-(hydroxymethyl)propane-1,3-diol
AUC: analytical ultracentrifugation
CTE: C-terminal extension
PDCD4: programmed cell death protein 4.
